# RAGE is a potential biomarker implicated in immune infiltrates and cellular senescence in lung adenocarcinoma

**DOI:** 10.1002/jcla.24382

**Published:** 2022-03-31

**Authors:** Zhihui Lin, Biyun Yu, Li Yuan, Jinjing Tu, Chuan Shao, Yaodong Tang

**Affiliations:** ^1^ Department of Pulmonary and Critical Care Medicine Ningbo Medical Center Lihuili Hospital Ningbo China

**Keywords:** bioinformatics, immune infiltration, lung adenocarcinoma, RAGE, senescence

## Abstract

**Background:**

Receptor for Advanced Glycation End‐products (RAGE) is an oncogene abnormally expressed in various cancers. However, the clinical value of RAGE and the biological role of RAGE in lung cancer have not been fully investigated.

**Methods:**

We compared the RAGE expression using several public databases. The relationship between RAGE expression and clinicopathological variables was assessed. The R software package was used to carry out enrichment analyses of RAGE co‐expression and gene set enrichment analysis (GSEA). Additionally, we used the TIMER database to assess the association between immune infiltration and RAGE expression. The correlation between RAGE expression and senescence biomarkers in lung adenocarcinoma was analyzed using the TCGA database.

**Results:**

Our findings indicated that the expression of RAGE was downregulated in lung adenocarcinoma, and down‐regulation of RAGE was related to poor overall survival and disease‐free survival. Functional enrichment analysis indicated that RAGE co‐expression genes were mainly associated with neutrophil activation involved in immune response, neutrophil degranulation, and regulation of leukocyte‐mediated immunity. Correlation analysis revealed that RAGE expression was closely related to the purity of the tumor and immune infiltration. GSEA indicated that the RAGE‐related differential genes were mainly enriched in senescence‐related pathways. Besides, the RAGE expression was significantly associated with senescence‐related genes.

**Conclusion:**

Down‐regulation of RAGE expression was associated with poor prognosis, as well as defective immune infiltration and cellular senescence in lung adenocarcinoma.

## Graphical abstract

We compared the AGER expression using several public databases. The relationship between AGER expression and clinicopathological variables was assessed. R software package was used to carry out enrichment analyses of AGER co‐expression and gene set enrichment analysis (GSEA). Additionally, we used TIMER database to assess the association between immune infiltration and AGER expression. The correlation between AGER expression and senescence biomarkers in lung adenocarcinoma was analyzed using TCGA database.

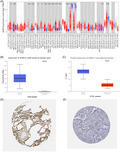

## 1 INTRODUCTION

In recent decades, lung cancer has been one of the most common cancers in the world and it accounted for the largest proportion of all tumor‐related deaths.^1^ Lung adenocarcinoma (LUAD) is the most prevalent subtype of non‐small cell lung cancer.^2^ Although non‐invasive surgical resection, immunotherapy, and immunotherapy have made great progress in recent decades, lung cancer treatment is still unsatisfactory, with a 5‐year relative overall survival rate of about 5%.^3^ Besides, LUAD is currently diagnosed in stages III and IV, when the tumor has spread to nearby organs and metastasized.^4^ Therefore, it is very necessary to screen more prognostic indicators and molecular pathological diagnosis of lung cancer.

Receptor for Advanced Glycation End‐products (RAGE), a transmembrane receptor of the immunoglobulin gene superfamily, is expressed in lung tissues and implicated in various diseases, including pulmonary disease, cancer, cardiovascular disease, autoimmune disease, and neurological disease.^5^ Previous reports have demonstrated that RAGE plays an important role in tumor development, such as pancreatic cancer, cervical cancer, and gastric cancer.^6^, ^7^, ^8^ lncRAGE exerted an inhibitory activity on lung cancer progression via targeting RAGE.^9^ Overexpression of RAGE inhibited the migration, invasion, and proliferation abilities of human non‐small cell lung cancer cell lines (H1299 cells), implying that it may serve as a therapeutic target for lung cancer.^10^ Moreover, RAGE was identified as a potential biomarker in lung cancer.^11^ It has been demonstrated that tumor immune‐microenvironment plays a vital role in untreated non‐small cell lung cancers.^12^ Senescence‐related secretion phenotype could regulate inflammatory response and contribute to immune infiltrates via generating lots of proteases, growth factors, chemokines, and cytokines, and further mediate tumor immune‐microenvironment.^13^ Cellular senescence is one of the primary risk factors for the pathogenesis of lung diseases, including lung cancer.^14^ To date, few types of research have investigated the correlation among RAGE expression, cellular senescence, and tumor immune infiltrates in lung cancer. In addition, further research still needs to be performed to illuminate the potential mechanism of RAGE in lung cancer.

In this research, we evaluated the expressions of RAGE in LUAD by using several databases. Furthermore, we investigated the correlation among tumor immune infiltrations, cellular senescence, and RAGE expression in LUAD, which offered a theoretical basis for exploring the potential molecular mechanisms.

## 2 METHODS AND MATERIALS

### 2.1 The Cancer Genome Atlas (TCGA) and Gene Expression Omnibus (GEO) database

TCGA database (www.tcga‐data.nci.nih.gov/tcga/) was used to collect the data of LUAD patients (515 tumor samples), including transcriptional expression data and matching clinicopathological characteristics. Besides, we also downloaded the mRNA expression profiling datasets (GSE1037, GSE43458, and GSE2088) from the GEO database to verify the RNA expression of RAGE between normal samples and tumor samples. The senescence‐related genes were collected from the GeneCards database. The differentially expressed genes (DEGs) between normal and tumor samples were collected by TCGA database, [log Fold Change (FC)] > 1 and *p *< 0.01 as the screening conditions.

### 2.2 Clinical Proteomic Tumor Analysis Consortium (CPTAC), UALCAN, and Human Protein Atlas (HPA)

CPTAC could provide protein analysis of each tumor sample.^15^ UALCAN is an interactive, user‐friendly, and comprehensive web for analyzing public data on various cancer.^16^ In the present study, UALCAN was used to analyze the RAGE mRNA expression from TCGA and the RAGE protein expression from CPTAC. The HPA contains lots of data on transcription and proteomics of human individual samples containing cell, tissue, and pathological profiles. It also contains protein immunohistochemistry data in human normal tissues and tumor tissues. In the present study, we compared the protein expression of RAGE between normal tissues and LUAD tissues.

### 2.3 Gene Expression Profiling Interactive Analysis (GEPIA) database

GEPIA database (http://gepia.cancer‐pku.cn/) could provide the analysis of gene expression profiling from 8587 normal samples and 9736 tumor samples in the GTEx and TCGA databases.^17^ In this study, we used this database to evaluate the relationship between survival and RAGE expression in LUAD. Besides, we used this database to further evaluate the correlation between co‐expressed genes and RAGE expression in LUAD.

### 2.4 Protein–protein interaction (PPI) network and functional enrichment analysis

The Search Tool for the Retrieval of Interacting Genes (STRING) is an online database that possesses a large amount of consolidated and integrated PPI data.^18^ In the present study, a PPI network of RAGE and co‐expressed genes was constructed by the STRING. The interaction score was set as >0.4. We used the “ClusterProfiler” package to carry out the Kyoto Encyclopedia of Genes and Genomes (KEGG) pathway and Gene Ontology (GO) enrichment analysis of co‐expressed genes, and the enrichment analysis results were visualized by the “ggplot2” package. LUAD data from TCGA were set into two groups based on the median expression of RAGE, and GSEA was performed using the clusterProfiler package. False discovery rate (FDR) <0.25 and p.adjust <0.05 were significance threshold.

### 2.5 Tumor immune estimation resource (TIMER) database

TIMER is an online database that contains 10,897 samples and 32 cancer types from the TCGA database. This database also can be used to systematically assess immune infiltrates in multiple cancer. In this research, we initially used the TIMER database to evaluate the RAGE expression levels from a pan‐cancer perspective. Then, this database was used to further investigate the correlation between immune cell infiltration (dendritic cell, neutrophil, macrophage, CD4+ T cells, CD8+ T cells, and B cells) and RAGE expression. Furthermore, the correlation between immune cell marker genes and RAGE expression was assessed by the TIMER tool.

### 2.6 Statistical analyses

R software was used to perform all statistical analyses. Mann–Whitney U‐test and paired t‐test were used to evaluate the differential expression level of RAGE between normal samples and LUAD samples. The ROC curve was visualized by using the pROC package. The correlations between RAGE expression and co‐expressed genes were analyzed using Spearman's correlation. *P* < 0.05 was the significance threshold.

## 3 RESULTS

### 3.1 Transcriptional levels of RAGE in LUAD patients

**FIGURE 1:**
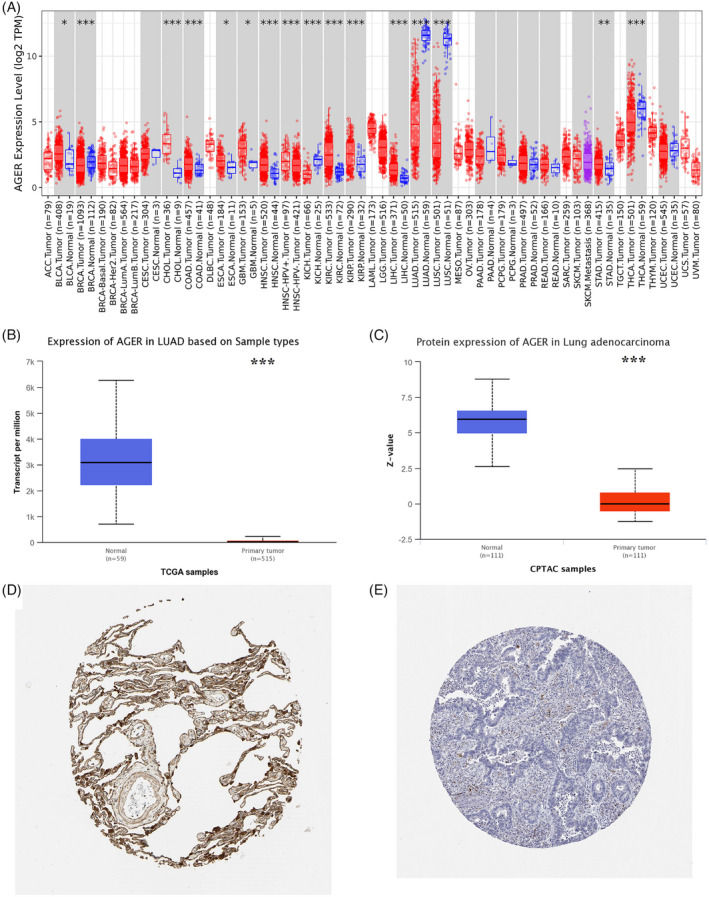
Expression levels of RAGE (alias for *AGER* gene) in human cancers. (A) RAGE expression levels in pan‐cancer perspective. (B) mRNA expression levels of RAGE in 515 LUAD tissues and 59 normal tissues. (C) Protein expression levels of RAGE in 111 LUAD tissues and 111 normal tissues. (D) The protein expression of RAGE in a normal sample. The intensity was strong, staining was high, and quantity >75%. (E) The protein expression of RAGE in LUAD. The intensity was negative, staining was not detected, and quantity was none. HPA normal: https://www.proteinatlas.org/ENSG00000204305‐RAGE/tissue/lung#img; HPA tumor: https://www.proteinatlas.org/ENSG00000204305‐RAGE/pathology/lung+cancer#img. (*p < 0.05, **p < 0.01, ***p < 0.001)

**FIGURE 2:**
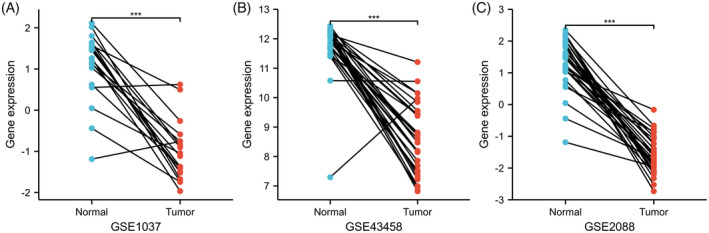
Validation of RAGE (alias for *AGER* gene) based on GEO database. (A) mRNA expression levels of RAGE in the GSE1037 dataset. (B) mRNA expression levels of RAGE in the GSE43458 dataset. (C) mRNA expression levels of RAGE in the GSE2088 dataset. ***p < 0.001

We used the TIMER database to compare the transcriptional levels of RAGE in normal and tumor tissues of various cancers. As shown in Figure 1A, compared with normal samples, RAGE is significantly downregulated in LUAD samples (*p* < 0.001). The TCGA database was used to further assess the mRNA expression of RAGE in LUAD. As shown in Figure 1B, compared with normal samples, the expression of RAGE is significantly downregulated in LUAD samples. Besides, the RAGE protein level is significantly lower in the LUAD samples than that in normal samples (Figure 1C). Furthermore, immunohistochemical staining from the HPA database indicated that RAGE protein expression is downregulated in LUAD samples (Figure 1D‐E). We also compared the RAGE expression between normal and tumor samples in GSE1037, GSE43458, and GSE2088 datasets (Figure 2A‐C), and the results were consistent with those mentioned above. In conclusion, these findings indicated that RAGE is downregulated in LUAD patients.

### 3.2 Relationships between clinical–pathological characteristics and RAGE mRNA levels

**FIGURE 3:**
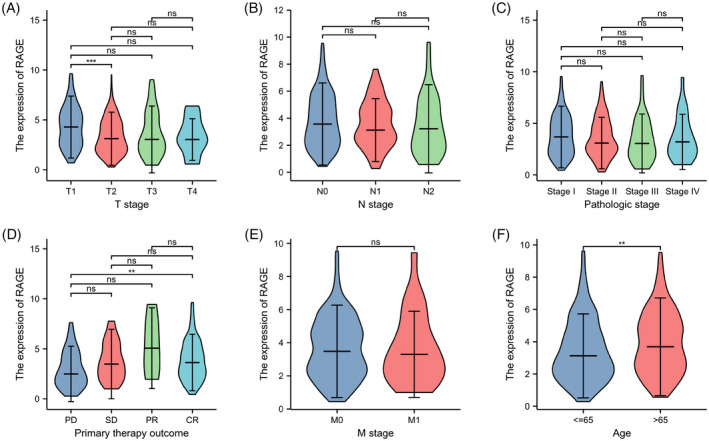
Relationships between clinical–pathological characteristics and RAGE (alias for *AGER* gene) mRNA levels. (A) RAGE expression is lower in the T2 stage than in the T1 stage, but no statistical difference is found in T3 and T4 stages. No statistical difference is found between RAGE expression and N stage (B), pathologic stage (C), primary therapy outcome (D), and M stage (E). (F) RAGE expression is lower in age <65 than in age >65

In our study, the relationships between RAGE expression levels and clinical–pathological characteristics of LUAD patients were evaluated to understand the relevance of RAGE expression in LUAD. As shown in Figure 3 and Table 1, compared with the T1 stage, RAGE expression significantly downregulated in the T2 stage (*p* < 0.001). There were no significant associations of RAGE expression with N stage, pathologic stage, primary therapy outcome, and M stage. Lower expression of RAGE is found in age <65 (*p* < 0.01).

**TABLE 1:** Clinical characteristics of LUAD patients between low expression of RAGE and high expression of RAGE

Characteristic	Low expression of RAGE	High expression of RAGE	*p*
*N*	267	268	
T stage, *n* (%)			
T1	66 (12.4%)	109 (20.5%)	0.002
T2	161 (30.3%)	128 (24.1%)	
T3	28 (5.3%)	21 (3.9%)	
T4	10 (1.9%)	9 (1.7%)	
N stage, *n* (%)			
N0	167 (32.2%)	181 (34.9%)	0.430
N1	51 (9.8%)	44 (8.5%)	
N2	38 (7.3%)	36 (6.9%)	
N3	2 (0.4%)	0 (0%)	
M stage, *n* (%)			
M0	174 (45.1%)	187 (48.4%)	0.872
M1	13 (3.4%)	12 (3.1%)	
Pathologic stage, *n* (%)			
Stage I	137 (26%)	157 (29.8%)	0.346
Stage II	68 (12.9%)	55 (10.4%)	
Stage III	45 (8.5%)	39 (7.4%)	
Stage IV	14 (2.7%)	12 (2.3%)	
Primary therapy outcome, *n* (%)			
PD	46 (10.3%)	25 (5.6%)	0.032
SD	17 (3.8%)	20 (4.5%)	
PR	2 (0.4%)	4 (0.9%)	
CR	155 (34.8%)	177 (39.7%)	
Gender, *n* (%)			
Female	137 (25.6%)	149 (27.9%)	0.364
Male	130 (24.3%)	119 (22.2%)	
Race, *n* (%)			
Asian	3 (0.6%)	4 (0.9%)	0.029
Black or African American	37 (7.9%)	18 (3.8%)	
White	195 (41.7%)	211 (45.1%)	
Age, *n* (%)			
<=65	144 (27.9%)	111 (21.5%)	0.004
>65	113 (21.9%)	148 (28.7%)	
OS event, *n* (%)			
Alive	151 (28.2%)	192 (35.9%)	<0.001
Dead	116 (21.7%)	76 (14.2%)	
DSS event, *n* (%)			
Alive	171 (34.3%)	208 (41.7%)	0.001
Dead	75 (15%)	45 (9%)	
PFI event, *n* (%)			
Alive	132 (24.7%)	177 (33.1%)	<0.001
Dead	135 (25.2%)	91 (17%)	
Age, median (IQR)	64 (58, 72)	67 (59, 74)	0.008

### 3.3 Differentially expressed of RAGE as a potential biomarker to identify LUAD samples

**FIGURE 4:**
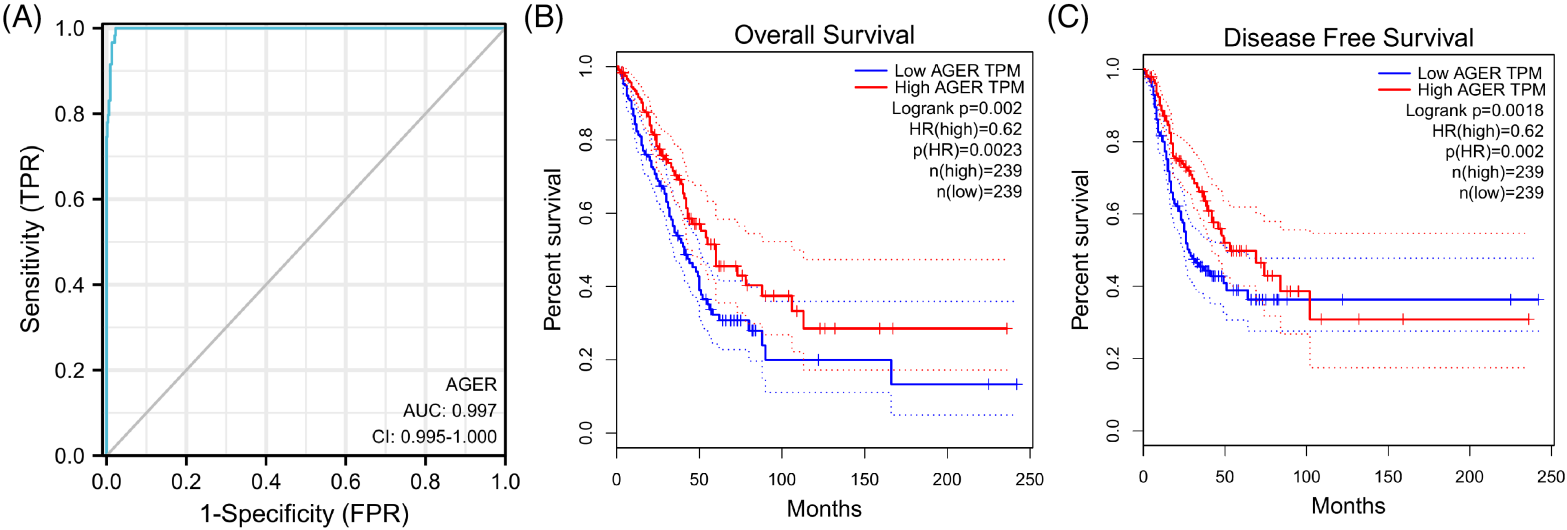
ROC curve and Kaplan–Meier curves for RAGE (alias for *AGER* gene) in LUAD. (A) ROC curve indicated that RAGE exhibited an AUC value of 0.997 to distinguish normal samples and LUAD samples. (B) Lower RAGE resulted in shorter overall survival. (C) Lower RAGE resulted in shorter disease‐free survival

The ROC analysis was performed to assess the diagnostic value of RAGE in LUAD. As shown in Figure 4A, the ROC curve was 0.997, indicating that RAGE exhibited a good diagnostic accuracy for LUAD. Besides, as shown in Figure 4B‐C, the results of overall survival and disease‐free survival indicated that low expression of RAGE in cancer patients predicated worse survival (*p* < 0.01).

### 3.4 RAGE co‐expression network in LUAD

**FIGURE 5:**
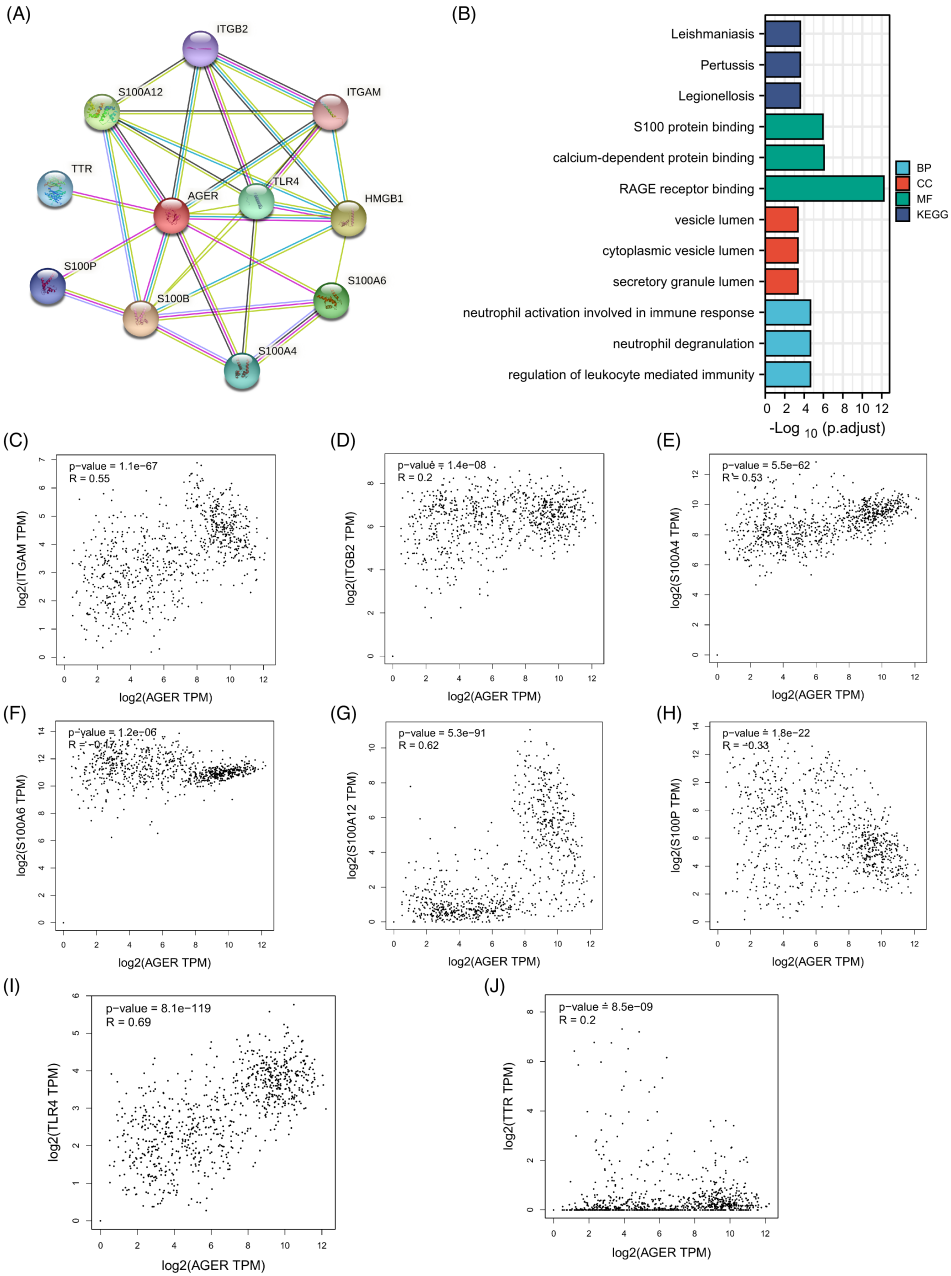
PPI network and functional enrichment analyses of RAGE (alias for *AGER* gene) co‐expression genes. (A) A PPI network of RAGE and its co‐expression genes. (B) Functional enrichment analyses of RAGE and its co‐expression genes. The correlation analysis of RAGE and its co‐expression genes: ITGAM (C), ITGB2 (D), S100A4 (E), S100A6 (F), S100A12 (G), S100P (H), TLR4 (I), and TTR (J)

In this research, we used the STRING database to structure the RAGE co‐expression network to further understand the biological functions of RAGE in LUAD. As shown in Figure 5A, the PPI network analysis indicated that RAGE is associated with TTR, S100P, S100B, S100A12, S100A4, S100A6, TLR4, HMGB1, ITGAM, and ITGB2. We also performed functional enrichment analysis of these co‐expression genes by using the R package clusterProfiler. As presented in Figure 5B, KEGG pathway analysis revealed that RAGE co‐expression genes were significantly enriched in Leishmaniasis, Pertussis, and Legionellosis. GO enrichment analysis indicated that RAGE co‐expression genes were mainly associated with neutrophil activation involved in immune response, neutrophil degranulation, regulation of leukocyte‐mediated immunity, S100 protein binding, calcium‐dependent protein binding, RAGE receptor binding, vesicle lumen, cytoplasmic vesicle lumen, and secretory granule lumen. Moreover, as shown in Figure 5C‐J, the correlation analysis showed that TTR, S100P, S100A12, S100A4, S100A6, TLR4, ITGAM, and ITGB2 have a significant correlation with RAGE. These findings revealed that RAGE plays an important role in the progress of LUAD.

### 3.5 Immune infiltration is associated with RAGE expression in LUAD

**FIGURE 6:**
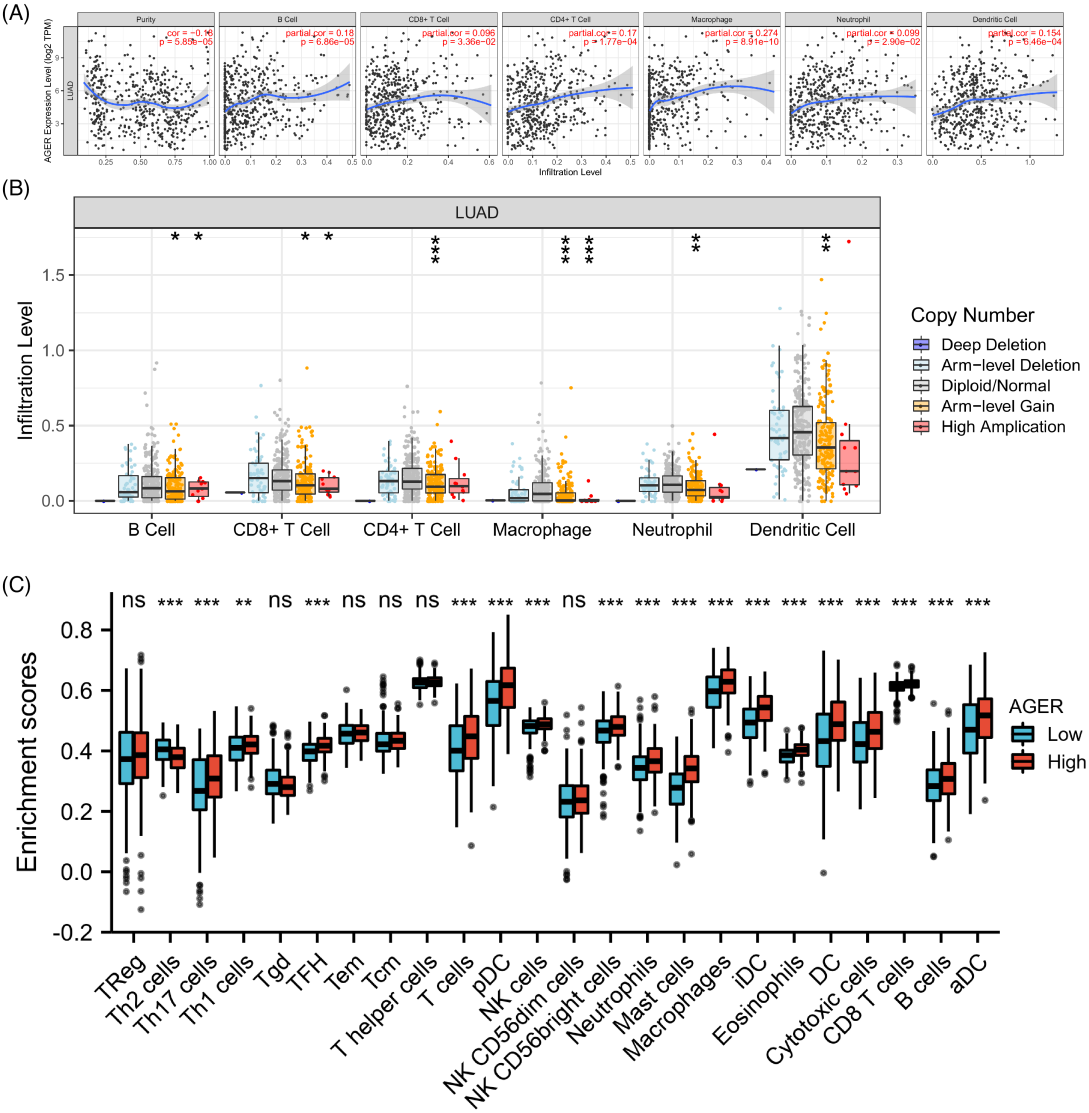
RAGE (alias for *AGER* gene) expression is associated with immune cell infiltrations in LUAD. (A) Correlation analysis between RAGE expression and immune cells. (B) The copy number variations of RAGE affects the infiltrating levels of dendritic cells, neutrophil, macrophages, CD4 + T cells, CD8 + T cells, and B cells in LUAD. (C) The differential expression of tumor‐infiltrating immune cells in high and low RAGE expression groups (ns: no significance, **p < 0.01, ***p < 0.001)

It has been demonstrated that tumor‐infiltrating lymphocytes affect the survival of cancer patients and are related to histopathologic and clinical risk factors in various cancers.^19^ Thus, we assessed the correlation between immune infiltration levels and RAGE expression in LUAD expression by using the TIMER database. As presented in Figure 6A, our findings showed that RAGE expression exhibited significantly positive correlation with infiltrating levels of dendritic cells (*p* = 6.46 × 10^−4^), neutrophil (*p* = 2.90 × 10^−2^), macrophages (*p* = 8.91 × 10^−10^), CD4 + T cells (*p* = 1.77 × 10^−4^), CD8 + T cells (*p* = 3.36 × 10^−2^), and B cells (*p* = 6.86 × 10^−5^). Moreover, we further assessed the immune infiltration levels in LUAD with copy number variations of RAGE. As shown in Figure 6B, the copy number variations of RAGE were significantly correlated with infiltrating levels of dendritic cells, neutrophil, macrophages, CD4 + T cells, CD8 + T cells, and B cells. These findings suggested that RAGE plays an important role in the immune infiltration of LUAD.

In our study, the differential expression of tumor‐infiltrating immune cells between the RAGE high‐expression and RAGE low‐expression groups was analyzed to access the role of the tumor immune microenvironment in LUAD. As shown in Figure 6C, the results indicated that the expression of Th2 cells, Th17 cells, Th1 cells, TFH, T cells, pDC, NK cells, NK CD56bright cells, neutrophils, mast cells, macrophages, iDC, eosinophils, DC, cytotoxic cells, CD8 T cells, B cells, and aDC were significantly different between RAGE low‐expression and RAGE high‐expression groups.

In this research, we also investigated the relationship between RAGE expression and multiple marker genes of tumor‐infiltrating immune cells by using the TIMER tool. As shown in Table 2, the marker gene CD19 of B cells, CD45 (PTPRC) of CD8+T cells, CD115 (CSF1R) of monocyte, BCL6 of Tfh, and STAT3 of Th17 are positively correlated with RAGE expression, which is positively correlated with all dendritic cells and M2 macrophage marker genes. Besides, some marker genes of T cells, M1 macrophage, neutrophil, Th1, Th2, and Treg are positively correlated with RAGE expression. These findings implied that RAGE expression is associated with immune cell infiltrations in LUAD.

**TABLE 2:** Correlation analysis between RAGE expression and markers of immune cells in TIMER

Description	Gene markers	Cor	*P*
B cells	CD19	0.1024	0.0230*
	CD79A	−0.0111	0.8054
	CD27	0.0193	0.6690
T cells	CD3D	0.0313	0.4882
	CD3E	0.1399	0.0018**
	CD2	0.1153	0.0104*
CD8 + T cells	CD8A	0.0489	0.2781
	CD8B	0.0192	0.6712
	CD45 (PTPRC)	0.1863	0.0000***
Monocyte	CD14	0.0093	0.8367
	CD86	0.0687	0.1275
	CD115 (CSF1R)	0.1167	0.0095**
M1 macrophage	INOS (NOS2)	0.1648	0.0002***
	IRF5	0.0925	0.0401*
	COX2 (PTGS2)	−0.0013	0.9764
M2 macrophage	CD163	0.1490	0.0009***
	CD206 (MRC1)	0.3015	0.0000***
Neutrophil	CD66b (CEACAM8)	0.3339	0.0000***
	CD11b (ITGAM)	0.1155	0.0102*
	CD15 (FUT4)	0.0128	0.7763
Dendritic cells	CD1C	0.3599	0.0000***
	CD141 (THBD)	0.3186	0.0000***
Th1	T‐bet (TBX21)	0.1663	0.0002***
	STAT4	0.1524	0.0007***
	IFNG	−0.1039	0.0211*
	TNF	0.0245	0.5870
	STAT1	−0.0726	0.1075
Th2	GATA3	0.0072	0.8726
	STAT5A	0.1940	0.0000***
	STAT6	0.3104	0.0000***
Tfh	BCL6	0.1416	0.0016**
	IL21	−0.0160	0.7231
Th17	STAT3	0.1032	0.0220 *
	IL17A	0.0766	0.0892
Treg	FOXP3	−0.0472	0.2959
	IL2RA	−0.0740	0.1006
	CCR8	−0.0024	0.9571
	TGFB1	0.1270	0.0047**
	STAT5B	0.1730	0.0001***

* *p* < 0.05, ^**^ *p* < 0.01, ^***^ *p* < 0.001.

### 3.6 Predicted the potential functions of RAGE in LUAD

**FIGURE 7:**
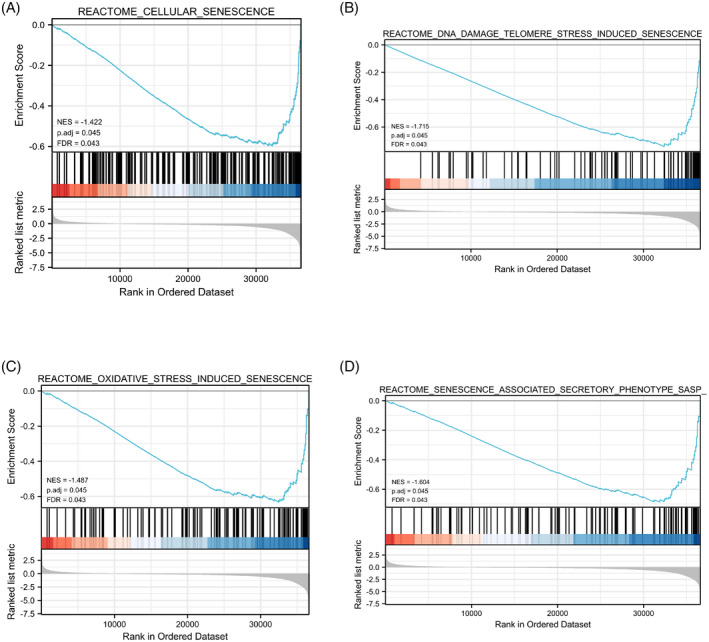
Gene set enrichment analysis (GSEA). The RAGE‐related differential genes were mainly enriched in (A) cellular senescence, (B) DNA damage telomere stress‐induced senescence, (C) oxidative stress‐induced senescence, and (D) senescence‐associated secretory phenotype

As shown in Figure 7, the results of GSEA revealed that the RAGE‐related differential genes were mainly enriched in senescence‐related pathways, such as cellular senescence, DNA damage telomere stress‐induced senescence, oxidative stress‐induced senescence, and senescence‐associated secretory phenotype.

### 3.7 Identification of cellular senescence‐related genes in LUAD

**FIGURE 8:**
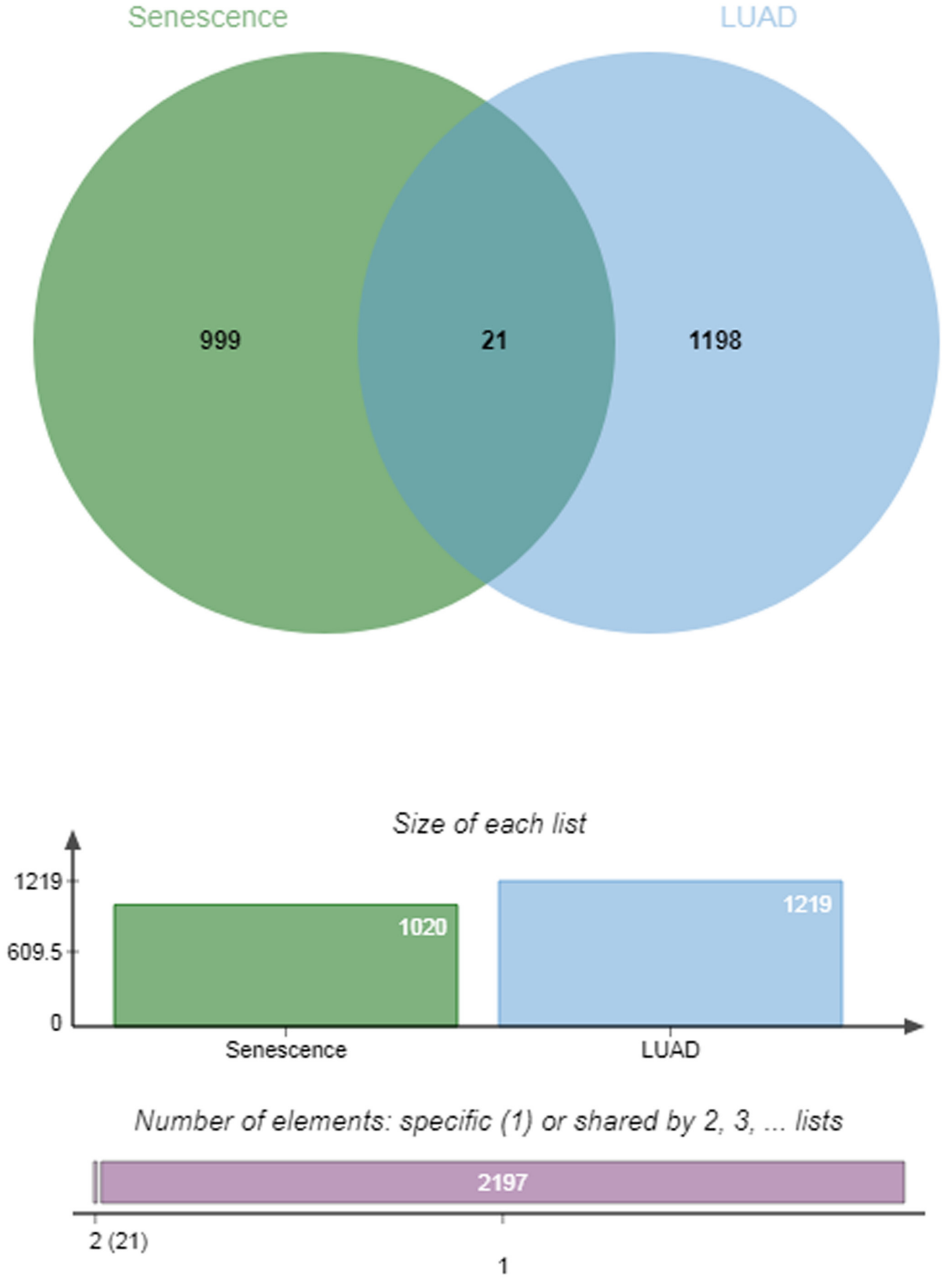
Venn diagram exhibiting the collective targets between senescence and LUAD

As shown in Figure 8, a total of 21 senescence‐related genes (SLC30A10, ECRG4, H4C1, MIR31HG, H1‐5, H1‐1, H2AC4, H2BC3, H2AC14, H2BC9, H4C13, H4C6, H2BC14, H4C4, H2BC10, H2BC7, H3C7, H3C11, RAGE, IGFBP1, and SERPINB5) were identified by overlap analysis.

### 3.8 RAGE expression is associated with cellular senescence in LUAD

**FIGURE 9:**
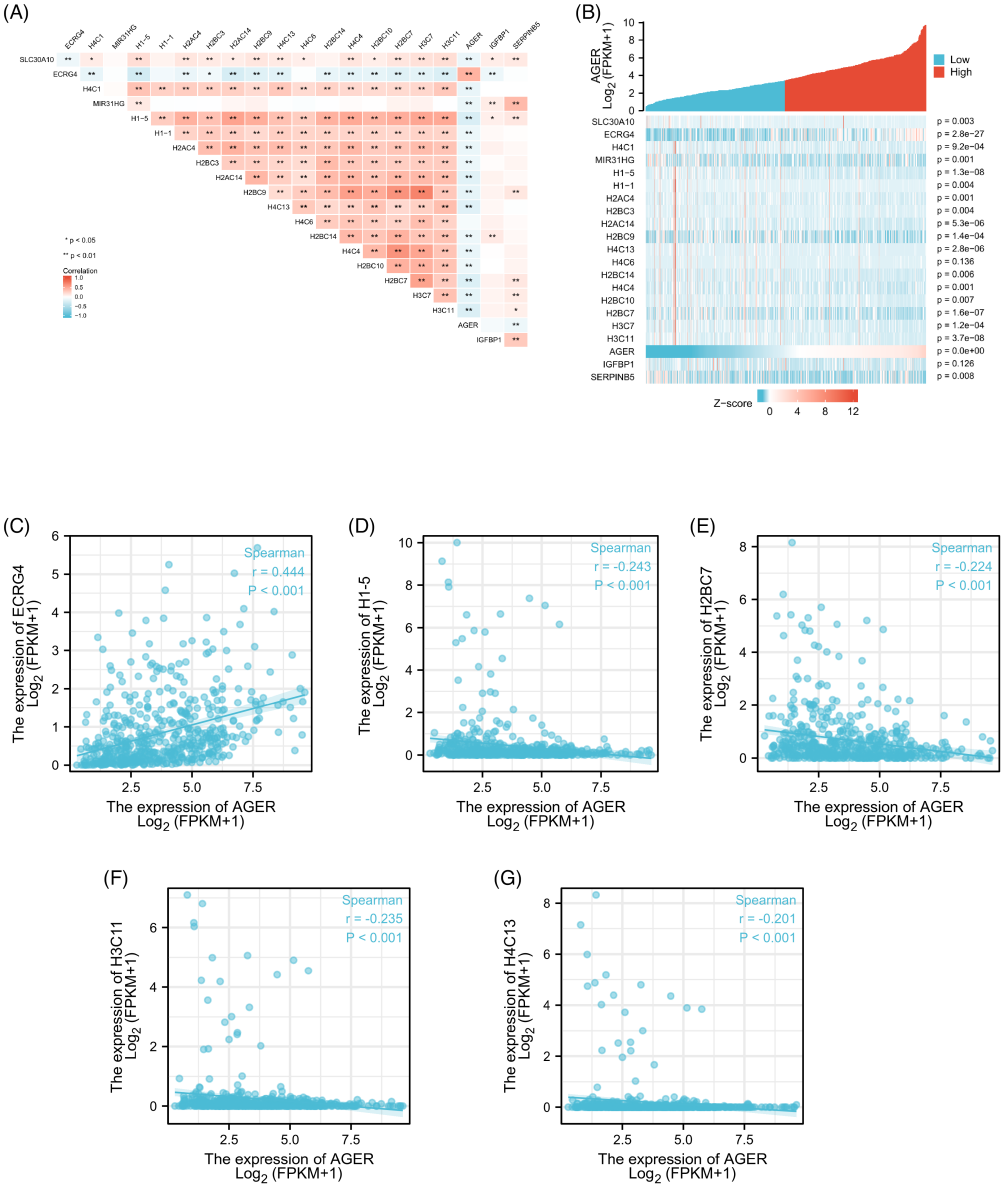
Correlation between RAGE (alias for *AGER* gene) expression and senescence‐related genes in LUAD. (A) Heatmap of 21 senescence‐related genes in LUAD. (B) Heatmap of the 21 senescence‐related genes between low and high RAGE expression groups. Draw a scatter plot to exhibit the correlation between RAGE expression and senescence‐related genes, such as ECRG4 (C), H1‐5 (D), H2BC7 (E), H3C11 (F), and H4C13 (G)

Cellular senescence has been reported to contribute to age‐related pathologies and tumorigenesis via the senescence‐associated secretory phenotype. In the present study, TCGA LUAD data were analyzed to assess the correlation between RAGE expression and 21 senescence‐related genes expression. We evaluated possible correlations between 21 senescence‐related genes in LUAD (Figure 9A). Figure 9B exhibited the heatmap of the 21 senescence‐related genes in the low‐level and high‐level RAGE expression groups. These results showed that expression of RAGE was significantly correlated with most senescence‐associated genes, except for IGFBP1 and H4C6. As displayed in Figure 9C‐G, the scatter plot indicated that RAGE was positively correlated with ECRG4 (r = 0.444, *p* < 0.001), while RAGE expression was negatively correlated with H1‐5 (r = −0.243, *p* < 0.001), H2BC7 (r = −0.224, *p* < 0.001), H3C11 (r = −0.235, *p* < 0.001), and H4C13 (r = −0.201, *p* < 0.001).

## 4 DISCUSSION

Lung cancer is one of the leading causes of cancer‐associated deaths worldwide.^20^ LUAD is the most common malignancy with a high risk of distant metastasis at each stage of disease.^21^ In recent years, great progress has been made in the treatment and diagnosis of LUAD. However, there is still a lack of effective diagnostic and prognostic approaches for patients with lung cancer in clinic.^22^ Thus, identifying novel prognostic biomarkers with effectiveness and high stability is beneficial to improve the prognosis of patients and implement precise treatment.

RAGE (alias for *AGER* gene) has been reported to participate in the pathophysiological process of various diseases, including cancers, pulmonary diseases, and neurodegenerative diseases.^5^ Up‐regulation of RAGE was identified as a biomarker for pneumonia and aggravated other lung diseases.^23^ Besides, a previous study has indicated that RAGE might be a potential biomarker associated with prognosis and cancer progression in LUAD.^24^ However, the underlying biological functions and mechanisms remain unclear.

The immune cells within the tumor microenvironment are involved in tumorigenesis.^25^ Recent researches have revealed that immune infiltration is associated with prognosis in all kinds of human cancers.^26^, ^27^, ^28^ It has been reported that uncontrollable immune dysfunction promotes the occurrence and development of cancer.^29^, ^30^ The inextricable link between lung cancer and immune infiltration has been repeatedly demonstrated.^31^, ^32^, ^33^ However, the association between immune infiltration and RAGE expression has not been reported. In the present study, GO‐BP enrichment analysis indicated that these co‐expressed genes of RAGE were implicated in the immune‐related pathways, including neutrophil activation involved in immune response, neutrophil degranulation, and regulation of leukocyte‐mediated immunity. Our findings indicated that RAGE expression was positively correlated with the infiltration of dendritic cells, neutrophil, macrophage, CD4+ T cells, CD8+ T cells, and B cells in LUAD. We also demonstrated that RAGE copy number alterations were associated with the infiltration level of dendritic cells, neutrophil, macrophages, CD4 + T cells, CD8 + T cells, and B cells. Furthermore, the levels of Th17 cells, TFH, T cells, pDC, NK cells, NK CD56bright cells, neutrophils, mast cells, macrophages, iDC, eosinophils, DC, cytotoxic cells, CD8 T cells, B cells, and aDC decreased in the RAGE low‐expression group. These results implied that a potential relationship exists between RAGE and immune cells infiltrations in LUAD.

Cellular senescence has been involved in various diseases progression, including tumor, wound healing, and aging.^34^ Recent studies have revealed that cellular senescence actively contributed to age‐related diseases and tumorigenesis via the senescence‐related secretory phenotype.^35^ It has been demonstrated that cellular senescence promoted carcinogenesis in patients with chronic obstructive pulmonary disease.^36^ Besides, membrane‐bound CD40L contributed to cellular senescence and activated senescence‐associated secretory phenotype in LUAD.^37^ However, the correlation between cellular senescence and RAGE expression has not been investigated. Another major finding of the present study was that RAGE expression was related to the cellular senescence of LUAD. In the present study, through the GSEA of RAGE, the results indicated that it was significantly associated with senescence‐related pathways, such as cellular senescence, DNA damage telomere stress‐induced senescence, oxidative stress‐induced senescence, and senescence‐associated secretory phenotype. Furthermore, RAGE expression was significantly correlated with senescence‐associated genes, such as ECRG4, H1‐5, H2BC7, H3C11, and H4C13. Our results indicated that RAGE was screened out as a senescence‐related biomarker.

## 5 CONCLUSION

Conclusively, our results for the first time indicated that RAGE expression is downregulated in LUAD, and associated with poor prognosis and defective immune cells infiltration. RAGE also is a novel gene regulating cellular senescence in LUAD. Therefore, RAGE is a potential prognosis biomarker in patients with LUAD.

## CONFLICT OF INTEREST

All the authors declared that there is no conflict of interest.

## AUTHOR CONTRIBUTIONS

Zhihui Lin was responsible for the writing of the study. Biyun Yu was responsible for designing the research. Li Yuan, Jinjing Tu, and Chuan Shao were responsible for analyzing the data from a public database. Yaodong Tang was responsible for reviewing a draft of the study and approved the final draft.

## Data Availability

All data are available from the corresponding author upon reasonable request.
